# A Narrative Review of Theranostics in Neuro-Oncology: Advancing Brain Tumor Diagnosis and Treatment Through Nuclear Medicine and Artificial Intelligence

**DOI:** 10.3390/ijms26157396

**Published:** 2025-07-31

**Authors:** Rafail C. Christodoulou, Platon S. Papageorgiou, Rafael Pitsillos, Amanda Woodward, Sokratis G. Papageorgiou, Elena E. Solomou, Michalis F. Georgiou

**Affiliations:** 1 Department of Radiology, Stanford University School of Medicine, Stanford, CA 94305, USA; awoodward@stanford.edu; 2 2nd Department of Orthopaedic Surgery and Traumatology, Aghia Sophia Pediatric General Hospital, Thivon 3 Street, 15772 Athens, Greece; pplaton24@gmail.com; 3 Neurophysiology Department, Cyprus Institute of Neurology and Genetics, Nicosia 2371, Cyprus; rafaelp@cing.ac.cy; 4 1st Department of Neurology, Medical School, National and Kapodistrian University of Athens, Eginition Hospital, 15772 Athens, Greece; sokpapa@med.uoa.gr; 5 Internal Medicine-Hematology, University of Patras Medical School, 26500 Rion, Greece; elenasolomou@hotmail.com; 6 Department of Radiology, Division of Nuclear Medicine, University of Miami, Miami, FL 33136, USA; mgeorgiou@med.miami.edu

**Keywords:** theranostics, neuroradiology, artificial intelligence, brain tumor treatment, nuclear medicine

## Abstract

This narrative review explores the integration of theranostics and artificial intelligence (AI) in neuro-oncology, addressing the urgent need for improved diagnostic and treatment strategies for brain tumors, including gliomas, meningiomas, and pediatric central nervous system neoplasms. A comprehensive literature search was conducted through PubMed, Scopus, and Embase for articles published between January 2020 and May 2025, focusing on recent clinical and preclinical advancements in personalized neuro-oncology. The review synthesizes evidence on novel theranostic agents—such as Lu-177-based radiopharmaceuticals, CXCR4-targeted PET tracers, and multifunctional nanoparticles—and highlights the role of AI in enhancing tumor detection, segmentation, and treatment planning through advanced imaging analysis, radiogenomics, and predictive modeling. Key findings include the emergence of nanotheranostics for targeted drug delivery and real-time monitoring, the application of AI-driven algorithms for improved image interpretation and therapy guidance, and the identification of current limitations such as data standardization, regulatory challenges, and limited multicenter validation. The review concludes that the convergence of AI and theranostic technologies holds significant promise for advancing precision medicine in neuro-oncology, but emphasizes the need for collaborative, multidisciplinary research to overcome existing barriers and enable widespread clinical adoption.

## 1. Introduction

Neuro-oncology is a rapidly evolving field, marked by groundbreaking advancements and innovations in recent years. Despite the extensive research efforts that have been made, survival rates for brain and central nervous system (CNS) tumors remain poor. According to the latest (fifth edition) World Health Organization Classification of Tumors of the CNS, neoplasms are subdivided into more than a hundred distinct entities, defined by their genetic/molecular, histological, and clinical features [1]. Malignant tumors comprise approximately 30% of CNS neoplasms, with adult-type diffuse gliomas representing the most common subtype of primary malignant CNS lesions, accounting for a substantial annual mortality of nearly 20,000 individuals in the United States [2]. Other common CNS neoplasms include meningiomas, representing approximately one-third of primary brain tumors, pediatric brain tumors such as pediatric-type diffuse gliomas, and brain metastases, mostly originating from lung cancer, melanoma, or breast cancer [3]. This broad molecular and clinical heterogeneity of CNS tumors presents significant challenges for their accurate detection and effective management.

Driven by the urgent need for more effective therapies and a growing molecular understanding of tumor progression, the field has seen crucial developments in diagnostic and treatment strategies, forming the foundation of theranostics [4]. This term implies the integration of diagnostic with therapeutic approaches, mainly exploited by nuclear medicine, where a radiolabeled ligand, designed to target molecules highly expressed on tumor cells specifically, is utilized to detect and eradicate the neoplastic cells [4,5,6]. Radioactive iodine has historically been the primary agent to detect and eliminate malignant thyroid cells. Currently, the focus of theranostics is increasingly directed to neuroendocrine tumors, by leveraging the high levels of somatostatin receptor (SSTR) expression, conjugating a peptide that binds to the receptor with a radionuclide, forming Lu-DOTATATE, and in prostate cancer, by employing the prostate-specific membrane antigen (PSMA), conjugated with Lutetium-177 (Lu-PSMA) [7,8,9]. Lu-177 and Ga-68 are predominantly harnessed for this purpose, as they emit β-radiation capable of exerting cytotoxic effects on targeted tumor cells.

Several attempts have been made to apply theranostics in neuro-oncology in recent years, mainly targeting gliomas and metastatic tumors. The main Positron Emission Tomography (PET) tracers actively used to diagnose glioma patients are: 18F-FET, 11C-MET, and 18F-DOPA [10]. In contrast, with inconsistent effectiveness, various existing tracers are currently applied in neuro-oncology, such as [18F] FDG. Peptide receptor radionuclide therapies (PRRT) have been extensively explored in meningiomas, demonstrating promising results in patients with grade I tumors (well-differentiated, low malignant potential), which highly express type 2 SSTR. Similar favorable outcomes are not observed in patients with grade 2 and 3 (high-grade) meningiomas [11,12].

Beyond molecular imaging offered by PET tracer advancements and targeted therapies achieved with the development of novel beta-and alpha-emitting radiotracers, recent Artificial Intelligence (AI) innovations are redefining the landscape of cancer imaging, allowing for more precise tumor detection. Hence, this enables improved treatment planning and enhanced monitoring of cancer staging and disease progression. Machine learning (ML) and deep learning (DL) are the two main subfields of artificial intelligence that have seen widespread adoption in healthcare. ML encompasses a broad range of algorithms such as decision trees, support vector machines, and ensemble methods, and it is trained to recognize patterns in a dataset and, consequently, make predictions and decisions on unknown data presented [13]. These models often rely on manually selected or engineered features and are generally more interpretable, making them suitable for structured clinical data. Conversely, DL technology, a subset of ML, is presented with raw input data and autonomously determines a set of hierarchical representations to predict and identify patterns in previously unseen datasets [14] DL employs multi-layered neural networks that excel in processing unstructured data such as medical images and have demonstrated superior performance in tasks like tumor segmentation and image classification in neuro-oncology. However, even though DL seems to excel in AI-assisted diagnostics, it requires larger datasets and higher computational power to be trained, and it is less interpretable, making it less transparent in its decision-making.

These tools have been widely utilized in neuro-oncology research to ultimately support various diagnostic and medical therapy functions. While still mainly in the research phase, several studies have demonstrated that AI integration in medical imaging can enhance the visual assessment of tumors, enabling a more precise detection by generating higher-resolution images. Morphometric analysis and advanced lesion quantification have also provided a more nuanced understanding of tumor morphology [15]. AI’s contribution to radiomics has also proven valuable, allowing a deeper understanding of tumor biology and characteristics by linking genetic and molecular data to macroscopic imaging (anatomical or functional) features [16,17].

Artificial intelligence has emerged as a valuable tool in advancing theranostic strategies for neuro-oncology in recent years, as numerous studies have investigated its potential applications and clinical value. Using AI tools to predict tracer uptake and guide therapy by integrating imaging biomarkers and molecular data could enhance theranostic applications in identifying and eradicating brain tumors [18]. Combining molecular imaging modalities (for instance, PET or SPECT) with AI methodologies improves the evaluation of tumor progression by providing comprehensive insights into tumor biology and behavior.

Despite these advancements, a significant gap persists in integrating AI into theranostic strategies for neuro-oncology. While various studies have independently explored molecular imaging or AI-based diagnostics, a comprehensive synthesis that links the recent developments in nuclear medicine with AI-driven tools for CNS tumor detection is lacking. Current literature does not adequately describe how AI technologies can be practically employed to enhance the diagnostic precision, therapeutic planning, and outcome prediction in neuro-oncology, particularly in PET and SPECT imaging and radiotherapy.

Hence, this narrative review aims to fill the gap by providing a focused, up-to-date overview of the theranostic approaches in neuro-oncology, emphasizing three key domains: (1) novel radiopharmaceutical agents introduced in the past five years (2) advancements in nuclear medicine imaging modalities for CNS tumor detection, and (3) the emerging role of AI in optimizing both diagnostic accuracies and treatment outcomes. By bridging these domains, our review seeks to clarify the clinical value of AI-enhanced theranostics and present the vast potential for clinical translation in this rapidly evolving field.

## 2. Materials and Methods

This study is a narrative review exploring the integration of theranostics and artificial intelligence (AI) in diagnosing and treating brain tumors, including gliomas, meningiomas, and pediatric CNS neoplasms. The review synthesizes evidence from both clinical and preclinical studies, focusing on recent advancements in personalized neuro-oncology.

A comprehensive literature search was performed in PubMed, Scopus, and Embase to identify relevant articles published between January 2020 and May 2025. The search was conducted with a clinical research librarian (A.W.), combining Medical Subject Headings (MeSH) and text words.

Keywords included: theranostics, radiotheranostics, PET-guided therapy, Lu-177, brain tumors, glioma, glioblastoma, meningioma, neuro-oncology, artificial intelligence, machine learning, deep learning, neural networks, and image processing.

**Figure 1:**
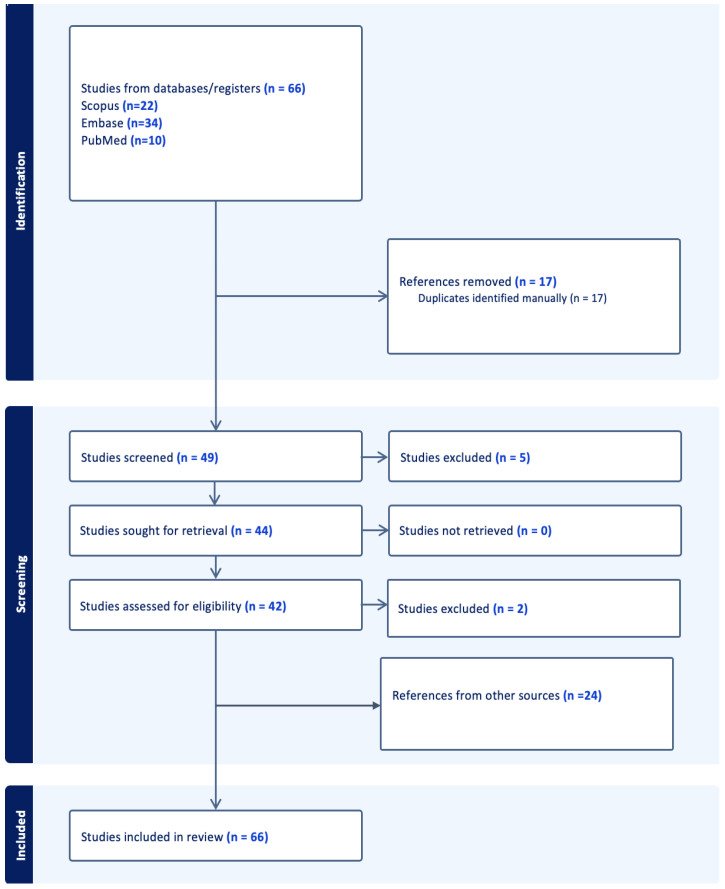
The workflow of search and selection. The search was conducted in May of 2025.

Articles were selected based on their thematic relevance to AI-enhanced theranostic approaches in neuro-oncology. Additional key studies were manually included to ensure completeness. The final manuscript cited 66 references. The workflow process can be found in Figure 1.

## 3. Results

Out of the 66 articles cited in this narrative review including those referenced for background and context a subset was thematically categorized into five core domains: (1) theranostic strategies in neuro-oncology, (2) artificial intelligence applications in neuro-oncologic imaging, (3) integration of AI with theranostic modalities, (4) current clinical and technical limitations, and (5) future directions. These thematic domains reflect the multidisciplinary evolution of precision diagnostics and AI-enhanced care in brain tumor management.

## 4. Discussion

### 4.1. Theranostics in Neuro-Oncology

#### 4.1.1. Overview of Theranostic Agents in Brain Tumors

Theranostic strategies in neuro-oncology aim to integrate diagnostic imaging with targeted therapy, and recent advancements in nanotheranostics have shown particular promise in gliomas. Emerging delivery strategies for targeted radionuclide therapy include pretargeting using avidin–biotin interactions, radiolabeled liposomes, and ligand-based systems. While pretargeting has shown promise in resectable tumors, and liposomes offer utility in identifying candidates for liposomal chemotherapy, ligand-based delivery remains the most promising approach. The ability to design and optimize ligands through molecular docking enhances receptor specificity and supports their growing role in theranostic agent development [19]. Some critical agents can be found in Table 1. For example, multifunctional nanocarriers can simultaneously deliver radioligand therapeutic agents and enable imaging via MRI, PET active nanocarriers with isotopes like ^64^Cu and ^99^mTc, or fluorescence, thereby allowing real-time treatment monitoring and precision targeting. For instance, magnetic nanoparticles such as SPIONs serve as MRI contrast agents and mediators of hyperthermia therapy. In parallel, novel cyanine dye-containing nanoparticles show promise in glioblastoma. These systems combine NIR-II fluorescence imaging with photothermal therapy (PTT) for real-time tumor visualization and ablation [19,20]. Strategies like AIE-induced steric hindrance have restricted intramolecular motion, increased molecular rigidity, and improved brightness. At the same time, ApoE peptide-functionalized liposomes enable active targeting of glioblastoma [20]. In addition, surface modifications with ligands such as transferrin or folic acid and gold PEGylated nanoparticles have enhanced transport across the blood–brain barrier (BBB). Compared to traditional contrast agents like SPIONs, which primarily support MRI and act through inducing hyperthermia, newer platforms such as NIR-II dye-based nanoparticles and functionalized liposomes offer multimodal capabilities by combining targeted delivery, enhanced imaging, and therapeutic functions. Thus, they represent a more integrated and precise approach to glioblastoma management.

Moreover, emerging techniques such as focused ultrasound combined with intravenous microbubbles can transiently disrupt the BBB, significantly improving the delivery and penetration of radiotracers and therapeutic agents—a significant challenge in glioma treatment [11]. It is worth mentioning that beyond the tumor itself, theranostics can target its microenvironment, such as tumor-associated myeloid cells (TAMCs). Notably, CD11b-expressing glioma-associated microglia/macrophages (GAMMs) have been successfully targeted in preclinical models, using a bifunctional anti-CD11b antibody conjugated with ^89^Zr (PET imaging) and ^177^Lu (therapy), leading to reduced TAMCs and enhanced immunotherapy outcomes [11]. This complementary strategy improves tumor visualization and addresses immunosuppressive components within the glioblastoma microenvironment, potentially enhancing the efficacy of immunotherapies.

Nanocostucts could also offer significant contributions to the field. They are composed of engineered nanoparticle assemblies and ligands, which are considered innovative theranostic platforms for precise delivery and real-time imaging in cancer care, by overcoming barriers such as off-target toxicity and drug release control, improving safety and treatment precision. These systems are being refined through AI-driven modeling into autonomous and nonautonomous [21]. This advancement allows the radiation oncology team to make preoperative decisions tailored to tumor complexity and anatomical location—determining whether to deploy a self-navigating nanoconstruct for difficult-to-reach or delicate regions, or an externally guided system for situations requiring precise control. Such personalization may lead to more effective treatments with fewer side effects and improved patient outcomes. Two recent studies have demonstrated the theranostic potential of CXCR4-targeted agents in high-grade gliomas. In the first study, a novel PET tracer, [(^68^Ga)] Ga-CXCR4, enabled precise tumor visualization in treatment-naive patients by emitting β-radiation while at the same time offering future utility for automated tumor delineation via machine learning [22]. In a second study, ^99^mTc-and ^177^Lu-labeled CXCR4 ligands (CXCR4-L) with nanomolar affinity were developed, confirming their theranostic potential in glioblastoma. These agents enable high-resolution imaging and targeted radiotherapy, with ^177^Lu-CXCR4-L emerging as a promising candidate for CXCR4-targeted radioligand therapy [23]. While both approaches utilize CXCR4 overexpression for tumor targeting, the first emphasizes diagnostic precision and potential for AI-driven image segmentation. In contrast, the second offers a more complete theranostic profile by combining SPECT imaging with radio-therapeutic delivery. Nevertheless, both studies are limited to preclinical or early-phase clinical stages, and key concerns such as off-target effects, long-term safety, and heterogeneous CXCR4 expression in gliomas remain to be fully addressed.

Additionally, given the involvement of the tumor microenvironment in gliomas, particularly the expression of fibroblast activation protein (FAP), the same radioisotope (^68^Ga) has been employed in the development of [^68^Ga] Ga-SMIC-3002. This FAP-targeted tracer, engineered with a quinolinium-based scaffold, exhibited high tumor specificity, strong in vivo stability, and significant uptake in FAP-expressing glioma tumors, further supporting its theranostic potential in neuro-oncology [24]. Despite tumor cells’ high uptake of the agent, significant accumulation was also observed in the liver and kidneys, highlighting the need for improved molecular design to enhance biospecificity and reduce off-target effects.

While theranostic research in neuro-oncology has focused mainly on gliomas, recent evidence supports using PSMA-targeted approaches in meningiomas. PSMA, typically associated with prostate cancer, was found to be expressed in nearly all meningioma specimens (98.9%), with higher levels correlating with tumor grade and recurrence. This endothelial expression persisted even after prior radiation, and quantitative PSMA metrics (e.g., PSMA/CD31 ratio) demonstrated predictive value for recurrence [25]. It could be a valuable biomarker for predicting survival in high-grade meningiomas and treatment therapy planning. Also, given that surgery may not be feasible due to factors like patient age, tumor localization, and co-morbidities, this theranostic approach could be a more effective alternative.

Tau (MAPT) has recently emerged as a novel glioma molecular target. Transcriptomic and proteomic analyses show that TAU protein is expressed in glioma cells, with higher levels in low-grade gliomas (LGGs) than glioblastomas (GBMs). Notably, high MAPT expression correlates with improved overall survival in LGG and GBM cohorts [26,27]. Given these findings, tau-targeted theranostic agents, such as radioiodinated Azure-A, which binds to aggregated tau and demonstrates excellent brain uptake with minimal toxicity, could serve as a dual-purpose imaging and treatment tool in a subset of gliomas [28]. These tools may be particularly valuable for preoperative tumor characterization, early relapse detection, or treatment response monitoring, especially in tau-enriched LGGs, where molecularly targeted approaches could offer a more personalized and less invasive alternative to conventional diagnostic and therapeutic strategies.

Furthermore, another promising theranostic strategy targets matrix metallopeptidase-2 (MMP2), overexpressed in many gliomas. A dendrimer-based nanoplatform utilizing chlorotoxin (CTX) for MMP2 targeting, conjugated with polyethylene glycol (PEG) and radiolabeled with iodine-131 (^131^I), has demonstrated high in vivo stability, selective tumor uptake, and dual-functionality for SPECT imaging and β-radiation therapy. This CTX-conjugated system represents a robust all-in-one platform for precisely diagnosing and treating MMP2-overexpressing gliomas [29].

**Table 1:** Emerging theranostic agents for brain tumors.

Agent/Approach	Target	Modality	Clinical Stage	Reference
177Lu-DOTATATE	Somatostatin Receptors	PET + Therapy	Approved	[6,28]
PSMA Radioligands	PSMA+ gliomas	PET	Investigational	[23,29]
CXCR4 Radioligands	CXCR4	SPECT/PET	Preclinical	[21]
Tau-targeting radiolabeled compounds	Tau protein in gliomas	SPECT	Preclinical	[26]
Gadolinium Nanoparticles	Tumor enhancement agents	MRI	Early Clinical	[30,31]
NIR-II Photothermal Nanoparticles	GBM cells	Optical + Thermal	Preclinical	[19]

#### 4.1.2. Evolving Clinical Applications of Theranostics

**Figure 2:**
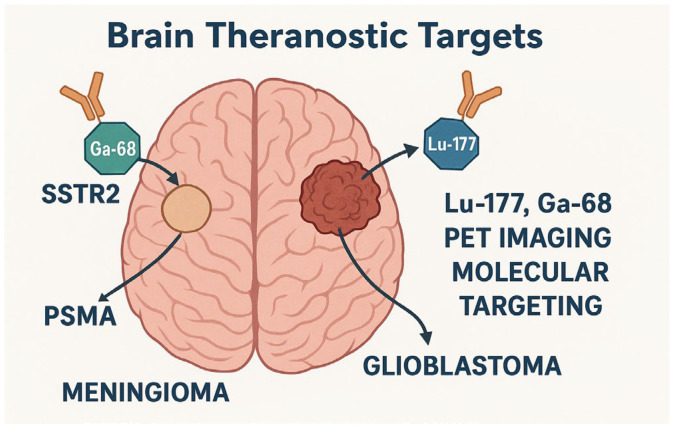
Summary of primary molecular targets and theranostic pairs in neuroradiology, featuring clinically relevant examples such as SSTR2 (^68^Ga-DOTATATE/^177^Lu-DOTATATE) and PSMA (^68^Ga-PSMA-11/^177^Lu-PSMA-617), as well as emerging targets and nanotheranostic approaches, with emphasis on the role of advanced imaging modalities and the need for personalized, multidisciplinary strategies.

Theranostic strategies have gained momentum in neuro-oncology, with several clinical applications demonstrating utility in gliomas and related CNS tumors (Figure 2). One of the most promising platforms involves Lu–177–based radionuclide therapies, which provide diagnostic and therapeutic capabilities. For instance, Lu-177-DOTATATE/DOTATOC has been evaluated for dose mapping and biodistribution, contributing important dosimetric data applicable to treatment planning in neuro-oncology, particularly in meningiomas, where somatostatin receptor expression supports its therapeutic application [30]. However, both agents share similar limitations, including potential renal toxicity and limited effectiveness in tumors with heterogeneous or low receptor expression. Their primary indication remains meningiomas and neuroendocrine tumors with high somatostatin receptor density, though ongoing research is evaluating their utility in other gliomas. Also, PSMA-targeting radioligand therapy, although traditionally used in prostate cancer, has been explored for brain tumors via intra-arterial administration, including meningiomas, where PSMA is expressed in tumor-associated vasculature. This could highlight the need to adapt existing tools through route-specific innovation [25,31].

Likewise, in the NANO-GBM trial, using AGuIX nanoparticles combined with standard radiotherapy and temozolomide in patients with newly diagnosed glioblastoma showed favorable safety outcomes and potential efficacy in enhancing radiosensitivity [32]. These gadolinium-based nanoparticles act as MRI contrast agents and radiosensitizers, representing a dual-function platform suitable for integration into standard clinical protocols. Preclinical data have shown that AGuIX gadolinium-based nanoparticles distribute heterogeneously but effectively within glioblastoma tissue after direct injection, supporting their role as theranostic agents [33]. These findings reinforce the clinical rationale behind the NANO-GBM trial exploring AGuIX with radiotherapy and temozolomide.

Another promising ^177^Lu-based therapy is currently in phase I/IIa, targeting gastrin-releasing peptide receptor (GRPR) in solid tumors. NeoB is a theranostic vector for GRPR-overexpressing gliomas, among other malignancies, marked as the first-in-human trial of this specific radioligand. The study aims to observe the effect that Lu-177-NeoB exhibits on therapy-resistant GBMs [3,34].

Paragangliomas, though rare in the CNS, have provided a model for applying targeted radionuclide therapy. In particular, therapies using ^177^Lu-DOTATATE and ^131^I-MIBG have been employed in cases of aggressive or unresectable paragangliomas due to their ability to selectively target somatostatin receptors and norepinephrine transporters, respectively [35]. These therapies extend survival and offer symptom palliation and tumor control, setting a precedent for similar approaches in gliomas with defined molecular targets. The conceptual framework of receptor-based targeting in paragangliomas supports the potential expansion of these principles into glioma subtypes expressing somatostatin receptors or other relevant molecular markers.

In pediatric brain tumors, theranostic strategies remain largely investigational, with limited clinically approved agents beyond ^^123^/^^131^I-mIBG for neuroblastoma. However, recent advances in radiolabeled nanoparticles and CXCR4-targeted imaging agents already under evaluation in adult gliomas suggest promising avenues for precise, low-toxicity diagnosis and therapy in pediatric neuro-oncology [36].

#### 4.1.3. Unmet Needs and Limitations in Neuro-Theranostics

Initially, despite the great potential of theranostic platforms in neuro-oncology, several limitations hinder their widespread clinical integration. Techniques like chemical exchange saturation transfer (CEST) MRI remain constrained by technical variability in pulse sequence settings and challenges in z-spectrum interpretation, limiting their reproducibility across centers [37]. Similarly, while AI-integrated nanorobots show potential for overcoming the blood–brain barrier (BBB), issues related to biocompatibility, precise in vivo navigation, and regulatory approval remain substantial hurdles [38]. The adverse effects of alpha and beta emitters remain insufficiently supported by robust clinical data.

### 4.2. Artificial Intelligence in Neuro-Oncology Imaging

#### 4.2.1. AI in Segmentation, Diagnosis, and Classification

Artificial intelligence (AI) has brought significant advancements to neuro-oncology, particularly in the imaging-based management of gliomas. The key applications are summarized in Table 2. Traditional diagnostic workflows—dependent on radiologist interpretation of multiparametric MRI and histopathological review—are limited by inter-observer variability and complex heterogeneity of brain tumors [39]. Machine learning (ML) and deep learning (DL) models now enable automated tumor segmentation, subtype classification, and molecular marker prediction (e.g., IDH mutation, 1p/19q co-deletion) using imaging alone. Deep learning architectures such as 3D U-Net, V-Net, and DeepMedic have demonstrated high accuracy in delineating tumor subregions and surrounding edema [40]. These models identify and accurately segment complex anatomical structures in three dimensions by learning hierarchical spatial features from volumetric medial images, using encoder–decoder structures and multi-scale processing. Notably, nnU-Net outperforms manual radiologist-based delineation, improving diagnostic precision and surgical planning. However, despite their strong performance, these models face significant limitations that challenge their clinical implementation. Most are trained on datasets from single institutions, which can introduce bias and reduce their generalizability to data acquired from different scanners or imaging protocols. Additionally, these models are prone to overfitting without adequate regularization or data augmentation, performing well on training data but less reliably on new, unseen cases. These challenges underscore the need for standardized, multicenter datasets and rigorous validation to ensure robust and reproducible performance across diverse clinical settings. Although certain limitations remain to be addressed, these tools hold significant potential for clinical integration, enhancing tumor detection and enabling more effective monitoring of disease progression.

In addition, a preclinical study introduced RMAP-ResNet, a residual multi-core attention pooling network for segmenting tumor tissue in optical coherence tomography (OCT) images. This model achieved a Dice coefficient of 94.78% on murine tumor data by leveraging attention mechanisms and adaptive receptive fields, demonstrating its potential for intraoperative tumor margin detection [47]. The significance of this approach lies in its application to OCT imaging, which is a challenging modality for delineating tumor boundaries. The model enables more precise and accurate segmentation by employing a technology that captures both the outlines and spatial distributions of target features. It integrates information from multiple receptive fields using an attention mechanism, applying parallel pooling layers with varied receptive fields. This architecture significantly enhances segmentation performance, particularly in low-contrast tumor OCT images, where previous models failed. However, the relatively small data sample and example pool must be considered when interpreting the high-performance metrics.

#### 4.2.2. Predictive Analytics: Prognosis and Response to Therapy

Beyond segmentation, AI enables predictive analytics for therapy planning. In a recent study on recurrent glioblastoma, researchers developed a spatio-temporal mathematical model integrating MRI-derived tumor burden and cellularity with SPECT/CT dosimetry to simulate tumor response to Rhenium–186–labeled nanoliposomes [18,48]. This model allowed delivery of highly localized radiation directly into the tumor bed, achieving significantly greater radiation dosing than conventional methods like CyberKnife or whole-brain radiotherapy. Such multimodal models could offer a framework for personalized therapy optimization. Moreover, MRI inputs integrated into machine learning models can predict genomic features and differentiate between low- and high-grade gliomas by identifying markers such as B2M, SRPX2, and SERPINH1. This approach holds the potential to improve prognostic accuracy significantly [49].

#### 4.2.3. AI for Radiomics and PET/CT Fusion Imaging

Radiomics and radiogenomics offer powerful noninvasive tools to link imaging features with tumor biology. More specifically, radiomics focuses on extracting quantitative features from medical images to describe tumor characteristics, such as shape, texture, or intensity. At the same time, radiogenomics aims to correlate these imaging aspects with underlying genetic or molecular profiles. Chiu et al. (2023) used multiparametric MRI to define 3D tumor habitats in glioblastoma, which machine learning models harness to characterize tumor heterogeneity and correlate with gene expression patterns [50]. Subregions such as edema and necrosis were associated with specific biological processes (e.g., VEGF expression, autophagy), highlighting spatial imaging biomarkers’ diagnostic and prognostic value. Advanced MRI combined with AI enables a noninvasive theranostic platform to link phenotypes with molecular targets, informing diagnosis and treatment. For example, these methods are gaining traction in glioblastomas as they can extract high-dimensional features from standard imaging modalities and correlate them with molecular alterations. Despite gliomas’ disappointing response to many targeted immunotherapies, partly due to tumor heterogeneity and an immunologically reduced microenvironment, these approaches could help develop more effective and targeted treatments [51]. Ongoing research shows optimistic results on preclinical imaging methods like cryo-imaging, which play a pivotal role in developing and validating theranostic agents for glioblastoma. One study used 3D whole-brain cryo-imaging of fluorescently labeled GBM in murine models to quantify tumor volume, vascular architecture, and dispersal patterns. Notably, glioma cells demonstrated significant perivascular spread, an essential feature of human GBM that complicates therapy and imaging interpretation [41]. Despite its high-resolution capabilities, 3D cryo-imaging is limited by its ex vivo nature, labor-intensive processing, and susceptibility to artifacts such as tissue shrinkage and GFP signal loss. These factors reduce their clinical reproducibility and highlight the need for more translational imaging approaches.

State-of-the-art PET/CT-MRI fusion imaging in neuro-oncology merges high-resolution anatomical detail with metabolic and molecular insights. PET tracers such as FDG, amino-acid, and somatostatin receptor ligands enhance lesion detection, treatment planning, and recurrence monitoring, while fusion with MRI guides precise targeting [52]. Integrating artificial intelligence elevates this fusion further: AI-driven algorithms are now used for multimodal image registration, tumor segmentation, and radiomic feature extraction from PET and CT datasets. Deep learning models like ISA-Net have explicitly been developed to segment tumors in PET CT images using spatial attention mechanisms, achieving high accuracy by leveraging complementary PET and CT features [43]. ISA-Net was validated in two separate datasets (STS and HECKTOR) with a five-fold cross-validation and compared to other state-of-the-art technologies. The model achieved 86% and 80% precision in segmentation performance, respectively. Additionally, AI applications in PET CT and MRI fusion have reduced segmentation time, enabled automated prognostic predictions, and improved workflow efficiency. These benefits are critical for integrating theranostic approaches into clinical practice [44].

SPECT is crucial in delivering theranostics and measuring the radiation dose needed in the pretreatment plan. However, its use comes with the limitation of lower spatial resolution compared to MRI or PET. To overcome the inherent spatial-resolution limitations of treatment-focused SPECT—especially for lesions smaller than 3 × the gamma-camera resolution—a novel AI-informed PET-guided reconstruction method, SPECTRE (Single Photon Emission Computed Theranostic Reconstruction), has recently been developed [45]. By using high-resolution PET data as anatomical and functional priors, SPECTRE leverages a hybrid kernelised expectation maximization algorithm to enhance SPECT reconstruction quality, resulting in PET equivalent resolution and improving accuracy in smaller lesions without noise. Notably, SPECTRE enables more accurate and personalized radiation treatment planning, especially in radionuclide therapy (RNT), by optimizing dose estimations and reducing volume segmentation errors compared to standard resolution-modeled SPECT. With an average volume difference from ground truth of only 26% using conventional fixed-threshold segmentation (versus 158% in standard OSEM), SPECTRE demonstrates how AI can transform theranostic imaging into a high-precision tool for therapy planning and monitoring [46].

Chemical Exchange Saturation Transfer (CEST) MRI enables non-invasive detection of endogenous molecules for brain tumor grading and assessment of IDH and MGMT status. Recent advances, including deep learning-based analysis, have enhanced its accuracy and theranostic potential. CEST offers label-free imaging, supports radiomics pipelines, and shows promise in tumor stratification and treatment monitoring [37].

#### 4.2.4. Challenges in Clinical Adoption of AI in Neuro-Oncology Imaging

Despite significant advancements in AI-driven imaging tools for glioma diagnosis and therapy planning, several barriers hinder their routine clinical integration. One of the most pressing issues is deep learning algorithms’ “black-box” nature, which limits interpretability and clinician trust [53]. Physicians are often reluctant to base critical decisions on predictions they cannot explain, especially in high-stakes settings like neuro-oncology. Moreover, many AI models are trained on single-institution or demographically narrow datasets, leading to poor generalizability when applied across diverse clinical environments [54]. This data heterogeneity challenges the scalability and reproducibility of model performance, which is essential for clinical utility. Regulatory and ethical concerns also pose hurdles; models must meet rigorous safety, efficacy, and transparency standards, particularly when applied in nuclear medicine or theranostic frameworks.

### 4.3. Integrating AI and Theranostics

#### 4.3.1. Synergy Between AI and Theranostics: Predicting Tracer Uptake and Guiding Therapy

Gliomas, particularly glioblastomas, are among the most aggressive brain tumors, with limited treatment efficacy due to heterogeneity, drug resistance, and poor penetration across the blood–brain barrier. Nanotheranostics, which integrates imaging-guided therapy and diagnostic functions, offers a promising approach by enabling targeted drug delivery and real-time treatment monitoring [21]. Accurate in vivo quantification of theranostic agents is essential for optimizing therapeutic efficacy and minimizing toxicity. A pre-clinical study showed that advanced MRI techniques, such as magnetization-prepared rapid gradient echo (MPRAGE) sequences, can now generate quantitative T1 maps as part of standard clinical protocols to assess concentrations of agents like gadolinium-based nanoparticles in metastases [55]. These imaging biomarkers can be integrated into AI algorithms to predict tracer uptake, enabling patient-specific dose planning and treatment adaptation. Additionally, radiotracers targeting chemokine receptor-4 (CXCR4), imaged using PET/CT, provide further opportunities for AI-assisted tumor delineation and therapy guidance [11,22,56]. Collectively, these strategies represent a powerful synergy between AI and theranostics, offering more precise and personalized treatment strategies for glioma patients.

#### 4.3.2. Precision Medicine: AI to Optimize Theranostic Protocols

**Figure 3:**
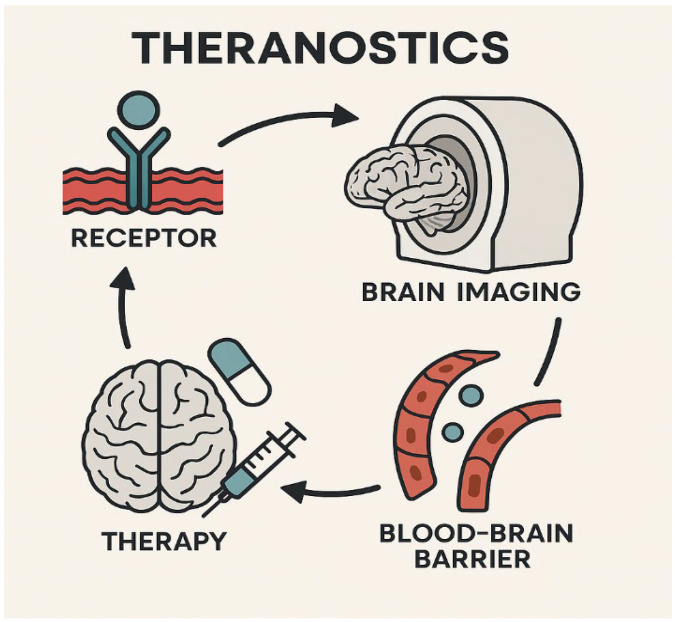
Illustration of the blood–brain barrier (BBB) structure and current strategies to enhance drug delivery in theranostic applications, including focused ultrasound, nanotheranostic platforms, convection-enhanced delivery, and intra-arterial administration, highlighting the challenges and solutions for effective brain tumor targeting.

Among the most significant challenges in neuro-oncology is achieving effective delivery of theranostic agents across the blood–brain barrier (BBB) Figure 3. Recent advances in nanotechnology and computational modeling have enabled the development of nanorobots capable of navigating the BBB and delivering payloads with high precision [57]. These nanocarriers are now engineered to respond to specific biochemical cues within the brain tumor microenvironment. Artificial intelligence and machine learning further enhance this field by enabling in silico design and nanorobot behavior simulation, helping predict delivery efficacy, minimize toxicity, and optimize pharmacokinetics [21]. AI-powered systems can tailor these platforms to individual patients by integrating imaging, molecular, and anatomical data, thus improving safety and clinical outcomes.

In parallel, clinical strategies such as super-selective intra-arterial administration of radiolabeled agents, exemplified by the use of [^68^Ga] Ga-PSMA-11 PET/MRI, have shown a 15-fold increase in tumor uptake compared to intravenous methods, with negligible off-target effects [31]. These data validate the concept of precision delivery and present new opportunities for AI to guide patient-specific administration strategies, integrate multi-modal imaging data, and enhance overall therapeutic outcomes.

#### 4.3.3. Role of Multimodal Imaging and AI to Enhance Treatment Planning

The demand for imaging studies to enhance diagnostic and treatment planning is gradually increasing in US hospitals annually. Likewise, in theranostics, PET, MRI, and SPECT, combined with artificial intelligence, could transform treatment precision. Many preclinical imaging studies have opened new avenues in the theranostic world. Radiomic and radiogenomic approaches in murine models allow for controlled investigation of the causal links between imaging characteristics and underlying molecular alterations [58]. At the same time, a novel paradigm known as radiovesicolomics is emerging, which combines extracellular vesicle (EV) profiling rich in molecular and radiomics, radiogenomics data, with radionuclide imaging techniques like PET and SPECT. Radiovesicolomics more specifically integrates the molecular cargo of EVs (such as proteins, RNAs, and lipids), which reflect the physiological and pathological state of tumor cells, with imaging-derived features to enable a more comprehensive characterization of tumors. This dual strategy leverages high-throughput input molecular insights from EVs with spatially resolved imaging, allowing more refined treatment targeting and real-time monitoring [59].

In addition to molecular imaging platforms, novel device-based theranostics such as ultracompact microneedles combining optical coherence tomography (OCT) and laser ablation are emerging [60]. While not molecular, these tools offer precise image-guided therapy in deep-brain tissue. When integrated with AI for image analysis and decision-making, they contribute to a broader multimodal theranostic strategy in neuro-oncology [15].

#### 4.3.4. Early-Phase Clinical Studies and Proof-of-Concept Applications in Theranostics

The theranostic landscape in neuro-oncology is advancing rapidly, with several clinical trials and pilot studies exploring applications in aggressive tumors such as glioblastoma. A notable strategy targets the tumor microenvironment, specifically CD11b^+^ myeloid cells, which promote immunosuppression in gliomas. A dual-modality agent using Zr-89 for PET imaging and Lu-177 for radioimmunotherapy successfully visualized and treated these immune cells in preclinical models, leading to tumor accumulation, immune modulation, and improved survival [61].

In parallel, innovative delivery frameworks are emerging. One recent approach integrates intra-arterial radiopharmaceutical delivery with molecular imaging, AI-based modeling, radiomics, and radiogenomic data to overcome drug delivery barriers in glioblastoma [62]. This strategy highlights the critical role of computational tools and personalized planning in advancing theranostics from concept to clinical application.

### 4.4. Current Gaps and Limitations

Despite significant progress in theranostic strategies within neuro-oncology, several critical limitations persist. A major hurdle is the limited number of clinical trials in neuro-theranostics, particularly regarding large-scale or multicenter studies. For example, the NANO-GBM trial, while promising, remains at the Phase 1b level and thus lacks the broad validation needed for clinical integration across diverse patient populations [32].

Interoperability and data standardization also remain unresolved challenges. Accurate quantification and dosimetry are essential for effective treatment planning, yet current SPECT-based theranostic methods still require refinement to ensure reproducible, standardized outputs across centers [45].

Furthermore, regulatory and ethical considerations limit the rapid adoption of theranostics in neuro-oncology. These approaches’ development and clinical implementation involve multiple complex steps, including rigorous evaluations of safety and efficacy, which are essential to protect patient well-being. Additional challenges include long approval timelines for novel radiopharmaceuticals and ethical concerns regarding informed patient consent, data privacy, and potential long-term effects of targeted radiation exposure. These factors necessitate a cautious, stepwise approach to integrating theranostics into routine clinical practice while maintaining patient safety and ensuring the proof-of-concept is thoroughly documented. As seen in the case of PSMA-targeting agents being explored for brain tumors, translating therapies approved for one cancer type into another involves complex approval pathways and safety assessments, slowing clinical deployment [25].

Future efforts must prioritize standardized imaging protocols, cross-institutional validation, and regulatory harmonization to fully harness theranostics’ promise in the brain.

### 4.5. Future Perspectives

The future of theranostics represents a paradigm shift toward highly personalized Table 3, AI-driven precision medicine, promising to revolutionize cancer treatment and patient care. Real-time artificial intelligence analytics will fundamentally transform next-generation theranostic approaches that enable dynamic treatment optimization. AI algorithms process vast datasets from imaging, genomics, and clinical records to provide unprecedented accuracy in tumor characterization, patient risk stratification, and response prediction [63]. Personalized AI-based theranostic pipelines will leverage machine learning to create individualized treatment protocols that adapt in real-time based on patient-specific molecular profiles, dosimetry calculations, and treatment responses, moving away from traditional one-size-fits-all approaches toward truly customized radiopharmaceutical therapies [64]. Digital twins and virtual treatment planning technologies could create dynamic, data-driven virtual representations of patients that continuously evolve alongside their human counterparts, enabling clinicians to simulate treatment scenarios, predict outcomes, and optimize treatment strategies before implementation [65].

The integration of radio genomics with molecular profiling will provide noninvasive imaging surrogates for genetic and molecular targets, allowing for real-time monitoring of tumor biology and treatment response while eliminating the need for repeated invasive biopsies [42,66]. These converging technologies will enable clinicians to “see what they treat and treat what they see” at an unprecedented molecular level, with AI facilitating the discovery of novel radiopharmaceutical targets and supporting the development of next-generation theranostic agents that promise to deliver more effective, safer, and truly personalized cancer treatments.

### 4.6. Limitations of the Study

While this review highlights promising advances in integrating theranostics and artificial intelligence in neuro-oncology, several limitations should be acknowledged. Firstly, many referenced studies are based on small sample sizes, single-center cohorts, or preclinical models, limiting generalizability. There is also a notable scarcity of large, multicenter datasets with harmonized imaging, genomic, and clinical annotations, essential for training and validating scalable AI-driven theranostic tools. These gaps must be addressed through collaborative, interdisciplinary research efforts to ensure reproducibility and real-world applicability.

## 5. Conclusions

This review highlights the transformative potential of combining artificial intelligence (AI) and theranostic technologies in neuro-oncology. AI enables more accurate tumor detection, characterization, and response monitoring through advanced image analysis, radiogenomics, and predictive modeling. Concurrently, theranostic agents—such as Lu-177-based radiopharmaceuticals, CXCR4-targeted PET tracers, and multifunctional nanoparticles—are emerging as dual-purpose tools for diagnosis and treatment. These innovations promise to enhance clinical precision, personalize therapeutic approaches, and improve outcomes for patients with brain tumors.

Despite encouraging progress, key challenges remain. The field is limited by a few clinical trials, a lack of multicenter validation, and concerns regarding regulatory frameworks, data interoperability, and ethical implementation. Pediatric tumors, rare CNS entities like paragangliomas, and meningiomas all present unique opportunities—but also highlight the need for tailored theranostic strategies.

Realizing the full impact of AI-integrated theranostics requires sustained investment in collaborative, multidisciplinary research that bridges the gap between bioengineering, computational science, and clinical neuro-oncology. A coordinated approach across academia, industry, and regulatory bodies is essential to validate, scale, and ethically implement these technologies in routine care. Such efforts may usher in a truly personalized, data-driven neuro-oncology era.

## Figures and Tables

**Table 2:** Key AI applications in neuro-oncology theranostics.

AI Application	Clinical Utility	Imaging Modality	AI Model Type	Reference(s)
Tumor segmentation	Precise tumor boundary detection	MRI, PET-CT	CNN, U-Net, EfficientNet	[38,39,41]
Genetic/molecular prediction	Predict MGMT, IDH status from imaging	MRI	Radiogenomics, XGBoost	[16,17,42]
Treatment response prediction	Forecast therapy outcomes	PET/MRI	ML models, deep learning	[13,37,40]
Radiotherapy planning assistance	Optimize dosing/target volume	MRI, PET	Explainable AI, Radiomics	[43,44,45]
Theranostic agent matching	Match tracers to biomarker profiles	PET-CT	Decision support AI	[28,29,46]

**Table 3:** Challenges and future directions.

Challenge	Implication	Potential Solutions
Blood–brain barrier	Limits drug delivery	AI-designed nanocarriers, focused ultrasound
Lack of large annotated datasets	Hinders ML/AI model development	Federated learning, multicenter collaborations
Inter-modality variability	Reduces reproducibility	Standardized imaging protocols, harmonization AI
Regulatory approval of AI systems	Slows clinical translation	Transparent validation pipelines, explainable AI

## Data Availability

No new data were created.

## Abbreviations

The following abbreviations are used in this manuscript:

AI	Artificial Intelligence
BBB	Blood–Brain Barrier
CNS	Central Nervous System
CNN	Convolutional Neural Network
CT	Computed Tomography
DL	Deep Learning
DNA	Deoxyribonucleic Acid
FAP	Fibroblast Activation Protein
FDG	Fluorodeoxyglucose
GBM	Glioblastoma Multiforme
HGG	High-Grade Glioma
IDH	Isocitrate Dehydrogenase
IHC	Immunohistochemistry
LITT	Laser Interstitial Thermal Therapy
LN	Lymph Node
LNM	Lymph Node Metastasis
Lu-177	Lutetium-177
MGMT	O6-Methylguanine-DNA Methyltransferase
MRI	Magnetic Resonance Imaging
NIR	Near-Infrared
NIR-II	Second Near-Infrared Window
OCT	Optical Coherence Tomography
PET	Positron Emission Tomography
PSMA	Prostate-Specific Membrane Antigen
RLT	Radioligand Therapy
SPECT	Single Photon Emission Computed Tomography
TAU	Tubulin-Associated Unit
WHO	World Health Organization

## References

[1] Louis D.N., Perry A., Wesseling P., Brat D.J., Cree I.A., Figarella-Branger D., Hawkins C., Ng H.K., Pfister S.M., Reifenberger G. (2021). The 2021 WHO Classification of Tumors of the Central Nervous System: A Summary. Neuro Oncol..

[2] Schaff L.R., Mellinghoff I.K. (2023). Glioblastoma and Other Primary Brain Malignancies in Adults: A Review. JAMA.

[3] Tolboom N., Verger A., Albert N.L., Fraioli F., Guedj E., Traub-Weidinger T., Gempt J., Pioch M., Lassmann M., Preusser M. (2024). Theranostics in Neurooncology: Heading Toward New Horizons. J. Nucl. Med..

[4] Lawal I.O., Abubakar S.O., Ndlovu H., Mokoala K.M.G., More S.S., Sathekge M.M. (2024). Advances in Radioligand Theranostics in Oncology. Mol. Diagn. Ther..

[5] Lecocq Q., De Vlaeminck Y., Hanssens H., D’Huyvetter M., Raes G., Goyvaerts C., Caveliers V., Lahoutte T., Xavier C., Vanhove C. (2019). Theranostics in Immuno-Oncology Using Nanobody Derivatives. Theranostics.

[6] Shooli H., Nemati R., Ahmadzadehfar H., Aboian M., Jafari E., Jokar N., Farzin H., Ebrahimi S.A., Movahedi M., Alejo C.D. (2021). Theranostics in Brain Tumors. PET Clin..

[7] Seifert R., Alberts I.L., Afshar-Oromieh A., Rahbar K. (2021). Prostate Cancer Theranostics. PET Clin..

[8] Ichikawa Y., Kobayashi N., Takano S., Kato I., Endo K., Inoue T. (2022). Neuroendocrine Tumor Theranostics. Cancer Sci..

[9] Mittra E.S. (2018). Neuroendocrine Tumor Therapy: 177Lu-DOTATATE. Am. J. Roentgenol..

[10] Galldiks N., Lohmann P., Friedrich M., Werner J.M., Stetter I., Wollring M.M., Hutterer M., Holzgreve A., Kocher M., Hinz R. (2024). PET Imaging of Gliomas: Status Quo and Quo Vadis?. Neuro Oncol..

[11] Hooper G.W., Ansari S., Johnson J.M., Ginat D.T. (2023). Advances in the Radiological Evaluation of and Theranostics for Glioblastoma. Cancers.

[12] Salgues B., Graillon T., Horowitz T., Chinot O., Padovani L., Taïeb D., Sans V., Carpentier A., Genin J., Le Bars D. (2022). Somatostatin Receptor Theranostics for Refractory Meningiomas. Curr. Oncol..

[13] Di Nunno V., Fordellone M., Minniti G., Asioli S., Conti A., Mazzatenta D., Iorio A., Pompucci A., Monfardini L., Carboni N. (2022). Machine Learning in Neuro-Oncology: Toward Novel Development Fields. J. Neurooncol..

[14] Dana D., Gadhiya S., St Surin L., Li D., Naaz F., Ali Q., Fatima A. (2018). Deep Learning in Drug Discovery and Medicine; Scratching the Surface. Molecules.

[15] Alongi P., Arnone A., Vultaggio V., Fraternali A., Versari A., Casali C., Bonanno L., Palaia R., Pasqualetti F., Fara A. (2024). Artificial Intelligence Analysis Using MRI and PET Imaging in Gliomas: A Narrative Review. Cancers.

[16] Hajianfar G., Shiri I., Maleki H., Oveisi N., Haghparast A., Abdollahi H., Sharifi M. (2019). Noninvasive O6 Methylguanine-DNA Methyltransferase Status Prediction in Glioblastoma Multiforme Cancer Using Magnetic Resonance Imaging Radiomics Features: Univariate and Multivariate Radiogenomics Analysis. World Neurosurg..

[17] Lohmann P., Meißner A.-K., Kocher M., Bauer E.K., Werner J.-M., Fink G.R., Delbridge C., Summer L., Stumpp P., Bauer S. (2020). Feature-Based PET/MRI Radiomics in Patients with Brain Tumors. Neuro Oncol. Adv..

[18] Christenson C., Wu C., Hormuth D.A., Huang S., Bao A., Brenner A., Allen B., Kung H.F., Yong W.H., Radishev V. (2023). Predicting the Spatio-Temporal Response of Recurrent Glioblastoma Treated with Rhenium-186 Labelled Nanoliposomes. Brain Multiphys..

[19] Poletto G., Cecchin D., Bartoletti P., Venturini F., Realdon N., Evangelista L. (2022). Radionuclide Delivery Strategies in Tumor Treatment: A Systematic Review. Curr. Issues Mol. Biol..

[20] Huang H., Li M., Gu J., Roy S., Jin J., Kuang T., Li Y., Zhang M., Shi C., Xia X. (2024). Bright NIR-II Emissive Cyanine Dye-Loaded Lipoprotein-Mimicking Nanoparticles for Fluorescence Imaging-Guided and Targeted NIR-II Photothermal Therapy of Subcutaneous Glioblastoma. J. Nanobiotechnol..

[21] Mishra S., Bhatt T., Kumar H., Jain R., Shilpi S., Jain V. (2023). Nanoconstructs for Theranostic Application in Cancer: Challenges and Strategies to Enhance the Delivery. Front. Pharmacol..

[22] Roustaei H., Norouzbeigi N., Vosoughi H., Aryana K. (2023). A Dataset of [(68)Ga]Ga-Pentixafor PET/CT Images of Patients with High-Grade Glioma. Data Brief..

[23] Ávila-Sánchez M., Ferro-Flores G., Jiménez-Mancilla N., Ocampo-García B., Bravo-Villegas G., Luna-Gutiérrez M., Juárez-Mercado K., Chiu L.-L., Meléndez-Alafort L., Santiago-Guarneros D. (2020). Synthesis and Preclinical Evaluation of the 99mTc-/177Lu-CXCR4-L Theranostic Pair for In Vivo Chemokine-4 Receptor-Specific Targeting. J. Radioanal. Nucl. Chem..

[24] Li L., Cao R., Chen K., Qu C., Qian K., Lin J., Liu Y., Zhang W., Liu L., Wang J. (2024). Development of an FAP-Targeted PET Probe Based on a Novel Quinolinium Molecular Scaffold. Bioconjug. Chem..

[25] Tubre T., Hacking S., Alexander A., Brickman A., Delalle I., Elinzano H., Yao X., Choi E., Agostini M., Gupta N. (2022). Prostate-Specific Membrane Antigen Expression in Meningioma: A Promising Theranostic Target. J. Neuropathol. Exp. Neurol..

[26] Callari M., Sola M., Magrin C., Rinaldi A., Bolis M., Paganetti P., Tagliabue E., Lau M., Gatti L., Baseggio L. (2023). Cancer-Specific Association Between Tau (MAPT) and Cellular Pathways, Clinical Outcome, and Drug Response. Sci. Data.

[27] Gargini R., Segura-Collar B., Herránz B., García-Escudero V., Romero-Bravo A., Núñez F.J., González-Cao M., Velasco G., Barbazán J., Vázquez-Barquero A. (2020). The IDH-TAU-EGFR Triad Defines the Neovascular Landscape of Diffuse Gliomas. Sci. Transl. Med..

[28] Abdelaziz G., Shamsel-Din H.A., Sarhan M.O., Gizawy M.A. (2020). Tau Protein Targeting Via Radioiodinated Azure A for Brain Theranostics: Radiolabeling, Molecular Docking, In Vitro and In Vivo Biological Evaluation. J. Label. Compd. Radiopharm..

[29] Zhao L., Zhu J., Cheng Y., Xiong Z., Tang Y., Guo L., Zhao H., Wu J., Song X., Wu J. (2015). Chlorotoxin-Conjugated Multifunctional Dendrimers Labeled with Radionuclide 131I for Single Photon Emission Computed Tomography Imaging and Radiotherapy of Gliomas. ACS Appl. Mater. Interfaces.

[30] Hänscheid H., Lapa C., Buck A.K., Lassmann M., Werner R.A. (2018). Dose Mapping after Endoradiotherapy with 177-Lu-DOTATATE/DOTATOC by a Single Measurement after 4 Days. J. Nucl. Med..

[31] Pruis I.J., Van Doormaal P.J., Balvers R.K., Van Den Bent M.J., Harteveld A.A., De Jong L.C., Jansen G.H., Futterer J.J., Van Dijk L.V., van Laarhoven H.W. (2024). Potential of PSMA-Targeting Radioligand Therapy for Malignant Primary and Secondary Brain Tumours Using Super-Selective Intra-Arterial Administration: A Single Centre, Open Label, Non-Randomised Prospective Imaging Study. eBioMedicine.

[32] Biau J., Durando X., Boux F., Molnar I., Moreau J., Leyrat B., Just N., Tessonnier L., Moisan A., Gligorov J. (2024). NANO-GBM Trial of AGuIX Nanoparticles with Radiotherapy and Temozolomide in the Treatment of Newly Diagnosed Glioblastoma: Phase 1b Outcomes and MRI-Based Biodistribution. Clin. Transl. Radiat. Oncol..

[33] Carmona A., Roudeau S., L’Homel B., Pouzoulet F., Bonnet-Boissinot S., Prezado Y., Desbrée A., Moubarek M.S., Curie S., Lemaire A. (2017). Heterogeneous Intratumoral Distribution of Gadolinium Nanoparticles Within U87 Human Glioblastoma Xenografts Unveiled by Micro-PIXE Imaging. Anal. Biochem..

[34] Advanced Accelerator Applications (2025). [177Lu]-NeoB in Patients with Advanced Solid Tumors and with [68Ga]-NeoB Lesion Uptake (NeoRay).

[35] Pacak K., Taieb D., Lin F.I., Jha A. (2024). Approach to the Patient: Concept and Application of Targeted Radiotherapy in the Paraganglioma Patient. J. Clin. Endocrinol. Metab..

[36] Poot A.J., Lam M.G.E.H., Van Noesel M.M. (2020). The Current Status and Future Potential of Theranostics to Diagnose and Treat Childhood Cancer. Front. Oncol..

[37] Huang J., Chen Z., Park S.W., Lai J.H.C., Chan K.W.Y. (2022). Molecular Imaging of Brain Tumors and Drug Delivery Using CEST MRI: Promises and Challenges. Pharmaceutics.

[38] Rai A., Shah K., Dewangan H.K. (2023). Review on the Artificial Intelligence-Based Nanorobotics Targeted Drug Delivery System for Brain-Specific Targeting. Curr. Pharm. Des..

[39] Khalighi S., Reddy K., Midya A., Pandav K.B., Madabhushi A., Abedalthagafi M. (2024). Artificial Intelligence in Neuro-Oncology: Advances and Challenges in Brain Tumor Diagnosis, Prognosis, and Precision Treatment. NPJ Precis. Onc..

[40] Preetha R., Jasmine Pemeena Priyadarsini M., Nisha J.S. (2025). Brain Tumor Segmentation Using Multi-Scale Attention U-Net with EfficientNetB4 Encoder for Enhanced MRI Analysis. Sci. Rep..

[41] Qutaish M.Q., Sullivant K.E., Burden-Gulley S.M., Lu H., Roy D., Wang J., Leikan S., Hitron A., Raza S., Bhadri V. (2012). Cryo-Image Analysis of Tumor Cell Migration, Invasion, and Dispersal in a Mouse Xenograft Model of Human Glioblastoma Multiforme. Mol. Imaging Biol..

[42] Zhuang Z., Lin J., Wan Z., Weng J., Yuan Z., Xie Y., Lu Q., Yang N., Chen C., Zhou J. (2024). Radiogenomic Profiling of Global DNA Methylation Associated with Molecular Phenotypes and Immune Features in Glioma. BMC Med..

[43] Huang Z., Zou S., Wang G., Chen Z., Shen H., Wang H., Chen Y., Chen J., Zhang K., Chen X. (2022). ISA-Net: Improved Spatial Attention Network for PET-CT Tumor Segmentation. Comput. Methods Programs Biomed..

[44] Yang D., Wang Y., Ma Y., Yang H. (2025). A Multi-Scale Interpretability-Based PET-CT Tumor Segmentation Method. Mathematics.

[45] Marquis H., Deidda D., Gillman A., Willowson K.P., Gholami Y., Hioki T., Tsushima Y., Tran-Gia J., Bailey D.L., Schmidtlein C.R. (2021). Theranostic SPECT Reconstruction for Improved Resolution: Application to Radionuclide Therapy Dosimetry. EJNMMI Phys..

[46] Marquis H., Willowson K.P., Schmidtlein C.R., Bailey D.L. (2023). Investigation and Optimization of PET-Guided SPECT Reconstructions for Improved Radionuclide Therapy Dosimetry Estimates. Front. Nucl. Med..

[47] Fan Y., Gao E., Liu S., Guo R., Dong G., Tang X., Mei Y., Liu C., Zhang Q., Chen W. (2024). RMAP-ResNet: Segmentation of Brain Tumor OCT Images Using Residual Multicore Attention Pooling Networks for Intelligent Minimally Invasive Theranostics. Biomed. Signal Process. Control.

[48] Phillips W.T., Goins B., Bao A., Vargas D., Ghaghada K., Salem R., Helfferich J., Negrete R., Mahdavi M., Phillips M.S. (2012). Rhenium-186 Liposomes as Convection-Enhanced Nanoparticle Brachytherapy for Treatment of Glioblastoma. Neuro Oncol..

[49] Yang P., Feng P., Tian G., Zhao G., Yuan G., Pan Y. (2025). Integrative Machine Learning and Bioinformatics Analysis Unveil Key Genes for Precise Glioma Classification and Prognosis Evaluation. Comput. Biol. Chem..

[50] Chiu F.Y., Yen Y. (2023). Imaging Biomarkers for Clinical Applications in Neuro-Oncology: Current Status and Future Perspectives. Biomark. Res..

[51] Sansone G., Vivori N., Vivori C., Di Stefano A.L., Picca A. (2022). Basic Premises: Searching for New Targets and Strategies in Diffuse Gliomas. Clin. Transl. Imaging.

[52] Timilehin O. (2025). PET-CT and MRI: A Powerful Combination for Brain Tumor Assessment. https://www.researchgate.net/publication/389279006_PET-CT_and_MRI_A_Powerful_Combination_for_Brain_Tumor_Assessment.

[53] Cui S., Traverso A., Niraula D., Zou J., Luo Y., Owen D., Lu W., Siddiqui S., Snyder K., Tsai K.L. (2023). Interpretable Artificial Intelligence in Radiology and Radiation Oncology. Br. J. Radiol..

[54] Saboury B., Bradshaw T., Boellaard R., Buvat I., Dutta J., Hatt M., Thomassen A., Nooijen P.T., Vinjamuri S., Bahri M.A. (2023). Artificial Intelligence in Nuclear Medicine: Opportunities, Challenges, and Responsibilities Toward a Trustworthy Ecosystem. J. Nucl. Med..

[55] Lavielle A., Boux F., Deborne J., Pinaud N., Dufort S., Verry C., Ferrand J., Menei P., Bernis G., Chérel M. (2023). T1 Mapping From MPRAGE Acquisitions: Application to the Measurement of the Concentration of Nanoparticles in Tumors for Theranostic Use. J. Magn. Reson. Imaging.

[56] Sahoo L., Paikray S.K., Tripathy N.S., Fernandes D., Dilnawaz F. (2025). Advancements in Nanotheranostics for Glioma Therapy. Naunyn Schmiedebergs Arch. Pharmacol..

[57] Singh A.V., Chandrasekar V., Janapareddy P., Mathews D.E., Laux P., Luch A., Saleh N., Valsesia A. (2021). Emerging Application of Nanorobotics and Artificial Intelligence to Cross the BBB: Advances in Design, Controlled Maneuvering, and Targeting of the Barriers. ACS Chem. Neurosci..

[58] Monti S., Truppa M.E., Albanese S., Mancini M. (2023). Radiomics and Radiogenomics in Preclinical Imaging on Murine Models: A Narrative Review. J. Pers. Med..

[59] Stępień E.Ł., Rząca C., Moskal P. (2022). Radiovesicolomics-New Approach in Medical Imaging. Front. Physiol..

[60] Yuan W., Chen D., Sarabia-Estrada R., Guerrero-Cázares H., Li D., Quiñones-Hinojosa A., Huang P., Xing L. (2020). Theranostic OCT Microneedle for Fast Ultrahigh-Resolution Deep-Brain Imaging and Efficient Laser Ablation In Vivo. Sci. Adv..

[61] Foster A., Nigam S., Tatum D.S., Raphael I., Xu J., Kumar R., Salem A., Kalas T., Karschnia P., Bette S. (2021). Novel Theranostic Agent for PET Imaging and Targeted Radiopharmaceutical Therapy of Tumour-Infiltrating Immune Cells in Glioma. eBioMedicine.

[62] Albert N.L., Le Rhun E., Minniti G., Mair M.J., Galldiks N., Tolboom N., Rushing E.J., Senetta R., Brandes A.A., Dhermain F. (2024). Translating the Theranostic Concept to Neuro-Oncology: Disrupting Barriers. Lancet Oncol..

[63] Ayalew B.D., Abdullah Khan S.M., Alemayehu Z.G., Teferi M.G., Aboye B.T., Abdalla A., Atnafie S., Ahmed Z., Oumer S., Ibrahim M. (2025). Role of Emerging Theranostic Technologies in Precision Oncology: Revolutionizing Cancer Diagnosis and Treatment. Oncologie.

[64] Davis L., Smith A.-L., Aldridge M.D., Foulkes J., Peet C., Wan S., Basu S., Capala J., Eslick E.M., Hacker T.A. (2020). Personalisation of Molecular Radiotherapy through Optimisation of Theragnostics. J. Pers. Med..

[65] Katsoulakis E., Wang Q., Wu H., Shahriyari L., Fletcher R., Liu J., Shaban-Nejad A., Vachharajani V., Massey S., Jones M. (2024). Digital Twins for Health: A Scoping Review. NPJ Digit. Med..

[66] Fathi Kazerooni A., Akbari H., Hu X., Bommineni V., Grigoriadis D., Toorens E., Stoyanova R., Han S., Kalpathy-Cramer J., Sood S. (2025). The Radiogenomic and Spatiogenomic Landscapes of Glioblastoma and Their Relationship to Oncogenic Drivers. Commun. Med..

